# Traits Defining Sow Lifetime Maternal Performance

**DOI:** 10.3390/ani12182451

**Published:** 2022-09-16

**Authors:** Laura Vargovic, Jo-Anne Harper, Kim L. Bunter

**Affiliations:** 1 Animal Genetics and Breeding Unit, A Joint Venture of NSW Primary Industries and the University of New England, University of New England, Armidale 2351, Australia; 2 Rivalea Australia Pty Ltd., Corowa 2646, Australia

**Keywords:** sow, piglet, condition, environment, welfare, lifetime performance

## Abstract

**Simple Summary:**

In pig production, the ability of a sow to produce and rear a high number of viable piglets that meet weight targets for a desired age at slaughter is the ultimate goal. To achieve this, both management and breeding programs have to be tailored and continuously optimized. Reproductive characteristics, maternal attributes, and the ability to maintain body condition are highly variable, both within sows and amongst sows. For individual sows, maternal performance alters with age, and the environmental factors have a large impact on sow performance. This study demonstrated that the sow influences the number of high-quality slaughter pigs produced, which is not solely related to variation in litter size. Accounting for the sow’s influence on the number of tail-enders in breeding programs could assist with producing more high-quality slaughter pigs.

**Abstract:**

Declining sow performance with increasing parity or an increase in the number of poor- quality pigs potentially impacts on farm productivity. This study investigated the phenotypic and genetic background of the sow’s influence on (i) the number of pigs not meeting the industry standards (tail-enders) and (ii) changes in performance with parity. Data were available for 3592 sows and their litters (13,976 litters) from a pig production system in NSW, Australia. The mean, standard deviation (SD), and slope for trait values over time were estimated for the sow characteristic traits: number of born-alive (NBA) and stillborn (SB) piglets and body condition of sow recorded with a caliper (CAL), along with maternal effects on piglet performance, represented by: average piglet birth weight (APBW), number of weaned piglets (WEAN), and tail-enders (TEND). Traits were analyzed in ASReml 4.2, by using an animal model. The number of tail-enders produced by a sow is a heritable trait, with a heritability estimate of 0.14 ± 0.04. Sow characteristics and maternal effects on piglet performance expressed by mean and slope had similar heritability estimates, ranging from 0.10 ± 0.03 to 0.38 ± 0.05, whereas estimates for SD traits were generally not different from zero. The latter suggests individual variability in sow characteristics or maternal performance between parities is largely not genetic in origin. This study demonstrated that more attention is required to identify contributions to the problem of tail-enders, and that slope traits could potentially be useful in the breeding program to maximize lifetime performance.

## 1. Introduction

Sows vary in reproductive characteristics (e.g., litter size, piglet birth weight), their ability to maintain body condition (e.g., caliper score), and the maternal attributes they provide to offspring in their care (e.g., maternal antibodies, milk supply), affecting piglet survival, numbers weaned, and piglet quality at weaning. All of these traits can be described as “maternal” performance attributes. Ultimately, maternal effects carry over to post-weaning progeny performance and the number of pigs per sow selected as future parents or sold as finishers [1,2]. The ability of a sow to produce and rear a high number of viable piglets that meet weight targets for a desired age at slaughter is the ultimate goal in pig production. Irrespective of the target slaughter weight, which differs across countries and production systems, both management and breeding programs have to be tailored and continuously optimized to achieve this goal [3,4]. Reproductive traits have been genetically improved over recent decades [5,6,7]. Whilst an improvement in reproductive traits is advantageous to increase slaughter pig numbers, a downside is an increase of within-litter or within-sow variation in some traits across parities [7]. Although within-litter variation is heritable and can be reduced with selection, a correlated response could reduce mean performance of the trait under selection [8], which is undesirable. High variability can also lead to extremes in performance, and potentially affects welfare [9]. Within-litter and within-sow variability can be investigated using metrics such as standard deviation, coefficient of variation, or variance as a measure of uniformity [10,11,12]. The impact of these changes through to slaughter pig age is poorly characterized. Further, in a breeding context, a decline in high-quality slaughter pigs with sow age/parity may not be adequately addressed with existing breeding goals.

Environmental factors, such as management, nutrition, season, or health status, also have a large impact on sow performance [13]. One of the most common non-genetic descriptors of the sow’s performance is parity. Each parity is affected by the prevailing impact of both the environment and the genetic merit of the sow. On average, gilts have smaller litter sizes with smaller piglets than sows, as uterine capacity is not fully developed [14]. Subsequently, with increasing parity, litter size increases [15]. However, increasing litter size with parity is potentially accompanied by a reduction in average piglet birth weight and increased number of stillborn piglets. Due to the unfavorable correlation between litter size (total or born alive) and piglet birth weight or viability, increases in litter size increase piglet mortality, leading to reduced improvements in the number of weaned piglets. In addition, the growth rate is lower for piglets with low birth weight, leading to more variation in pig weights within the contemporary groups [16,17].

This study investigated the genetic and phenotypic variation in sow performance that develops over time, which has implications for breeding programs, along with management interventions. This study had two objectives: (i) to quantify the production of poorer quality pigs at slaughter age as a sow trait and (ii) to investigate the variability in the change in reproductive and maternal performance over parities. The hypothesis was that a trait defining the production of poorer quality pigs at slaughter is heritable and that the performance over time varies within sows and between sows.

## 2. Materials and Methods

The data used in this study were obtained from an existing commercial database provided by a pig production system in NSW, Australia, and, as such, did not require any approval from an animal ethics committee. The data were available from one farm located in NSW, Australia, for a single line of pedigreed sows (N = 3592), routinely recorded for reproduction between 20 September 2014 and 31 December 2021. Sires were mated (single sire) at random to many dams. Gilts and mixed-parity sows were kept in separate group-housing systems during their gestation, followed by individual crates during lactation. All piglets were individually identified within 24 h from birth and were grown out to slaughter age on their birth farm. At the selection point, which coincided with the end of the performance test to slaughter age (approximately 5 months of age), pigs were recorded for the back-fat thickness, eye muscle depth, and weight.

### 2.1. Data Preparation

Data preparation was carried out using the R statistical programming language [18] in a two-step process. Firstly, dams of piglets with individual piglet mortality recorded were identified to be included in this study (N = 3592). The period for recording piglet mortality was between 1 January 2017 and 31 May 2021. Individual piglet mortality was subsequently used to construct traits reflecting a sow’s ability to produce and nurse piglets surviving until the selection point, coinciding with the end weight for finishers (hereafter called off-test). A piglet was considered alive at birth (i.e., to be included in the analysis) if any of the following information was available: birth weight or number of teats, recorded at birth; back-fat thickness, eye muscle depth, or the weight at the end of the off-test (N = 80,806 piglets from 6720 litters). Partly recorded litters (where not all live-born piglets were recorded) were not used for analyses. The complete information for all piglets was available for 3299 sows with 6462 litters. To calculate lifetime traits for sows, all reproductive records (N = 13, 976 litters) were obtained for the focus sows (N = 3592). Additional information on sow condition was available as a caliper reading [19]. These were measured on the mating day connected to litter records. In total, 10,293 caliper records across parities were available. 

#### Trait Definitions

The reproductive records available at each parity were number born-alive piglets (NBA), number stillborn piglets (SB), average birth weight of piglets (APBW), and the number of weaned piglets (WEAN). The number of piglets in a litter without an off-test record (weight, back-fat thickness, or eye muscle depth), but not recorded as dead by day 70, was defined to be the number of tail-enders (TEND). The threshold of 70 days represents movement of the pigs to new accommodations, after which individual mortality was not recorded. Given that all selection candidates exceeding 70 kg were recorded for performance, this trait therefore represented piglets that did not meet production standards, according to the farm protocol, due to the inadequate development/growth/health to slaughter age. The caliper trait (CAL) was the number of increments on a caliper [19], reflecting the sow’s body condition. Maternal effects on piglet performance are illustrated predominantly by the traits APBW, WEAN, and TEND. Conversely, NBA, SB, and CAL are considered characteristics of the sow, rather than as maternal effects. Nevertheless, these sow traits have implications for progeny performance.

To investigate variation in sow characteristics or maternal performance across parities, the above records were used to calculate new variables over parities. Each trait was re-defined as a mean (e.g., mNBA), standard deviation (e.g., sdNBA), or slope (e.g., NBAsl), with a single observation per trait per sow, calculated from data recorded over the sow lifetime. If a sow was culled, or mated to produce crossbred progeny, the subsequent information was not available. The performance of all sows commenced with parity-one data. Therefore, variation in the number of contributing parities predominantly reflected the number of parities a sow was retained for in the herd. The mean and standard deviation traits were based on raw data, whereas the slope traits were obtained from models correcting for other systematic effects, as described in Section 2.2.

### 2.2. The Analyses of the Maternal Performance Traits

The trajectory of trait values across parities was estimated using a linear mixed effect model in the R package “lme4” [20]. Random effects included sow and sow–parity interaction, to estimate the slope within sow. The slope value reflects the rate at which sow or maternal performance changes with the age/parity. All sows had a common intercept.

Systematic effects were fitted irrespective of their significance, for model consistency. The systematic effect common for each observed variable was contemporary group defined by farrowing year quarter (N = 30 levels), to account for managerial and seasonal differences. Each trait’s model included the number of records a sow had for each of the observed variables fitted as a linear covariate, to account for different numbers of parities recorded. The model for APBW included total born piglets fitted as a linear covariate. If a sow had only one observation for each trait, the record was included in the analyses in order to obtain the correct solutions for systematic effects, but the resulting trait value for slope was treated as missing.

### 2.3. Estimation of Variance Components and Correlations

The variance components for the mean, standard deviation, and slope traits were estimated using data from 3592 sows, which themselves were progeny of 304 sires and 2017 dams. The complete pedigree contained 568 sires and 3195 dams extended over four generations. Contemporary groups (N = 30 levels) defined by farrowing year/quarter were based on the first parity of farrowing in the original dataset. Each model consisted of the contemporary group (CG) and the number of observations (parities) when the observed trait was recorded, fitted as a linear covariate. The models for APBW included the mean total born piglets across parities, also fitted as a linear covariate. Parameter estimation for each trait was obtained by fitting a linear mixed effects animal model using restricted maximum likelihood procedures in ASReml 4.2 [21]. Estimates of heritability were obtained from univariate analyses fitting the systematic effects, as described earlier. Genetic and phenotypic correlations for the number of tail-enders (TEND) with the other traits were estimated only for traits with heritabilities significantly different from zero, using a series of bivariate analyses.

## 3. Results

Traits were grouped into two categories, representing reproductive characteristics of a sow (NBA, SB, and CAL) and the traits reflecting their maternal effects on piglet performance (APBW, WEAN, and TEND). The reasoning was that, in the case of sow characteristics, the piglet cannot affect NBA and SB, and CAL is measured directly on the sow. On the other hand, the sow dictates the reproductive level (NBA and SB) and the piglet attributes at birth (APBW), which are maternal effects for early growth and survival, represented by WEAN and TEND.

### 3.1. Phenotypic Data

Descriptive statistics for each trait are shown in Table 1. The across-parity phenotypes were only available for sows with more than one parity recorded. Mean values for born alive, stillborn, piglet birth weight, and weaned piglets were within the ranges of published values, and are not discussed further. The new trait, the number of tail-enders (TEND), had a relatively high mean ± SD value of 3.89 ± 2.56, representing a high proportion of NBA pigs not reaching end of test at the targets for age/weight. The coefficient of variation (CV) for TEND traits was high relative to NBA, suggesting the number of tail-enders was not simply proportional to NBA.

**Figure 1:**
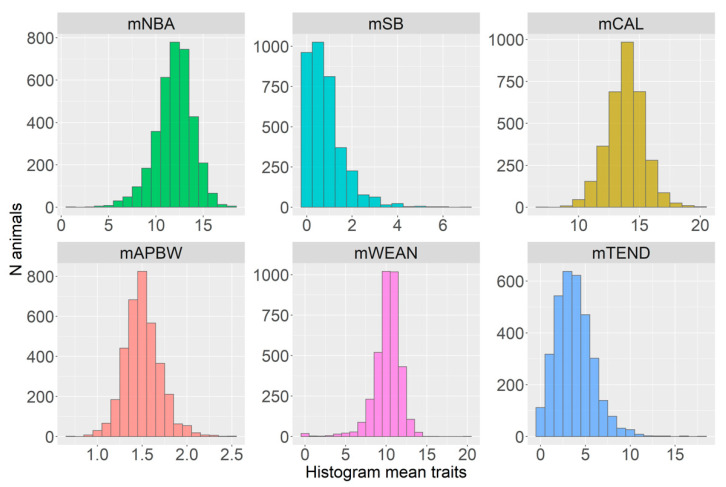
The distribution of mean traits (m*) for born-alive [NBA] and stillborn [SB] piglets, caliper [CAL]), average piglet birth weight [APBW], weaned piglets [WEAN], number of tail-enders [TEND].

**Figure 2:**
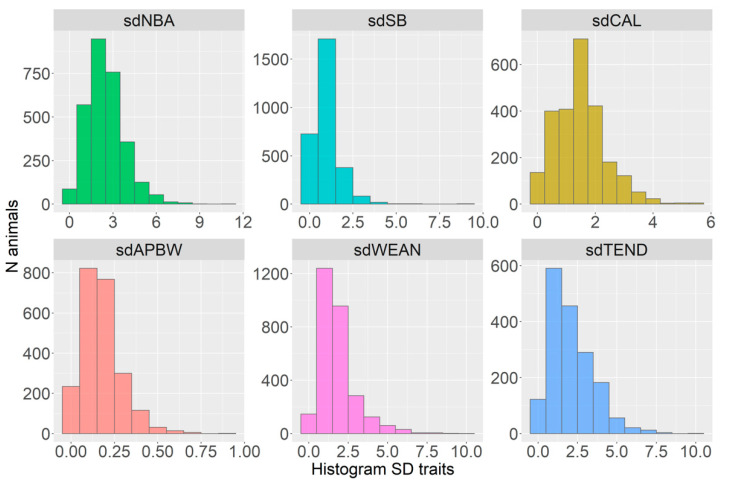
The distribution of SD traits (sd*) for born-alive [NBA] and stillborn [SB] piglets, caliper [CAL], average piglet birth weight [APBW], weaned piglets [WEAN], number of tail-enders [TEND].

**Figure 3:**
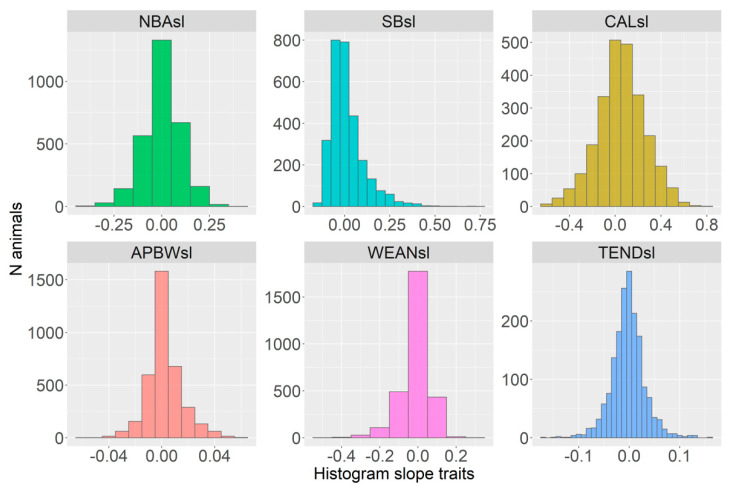
The distribution of slope traits (*sl) for born-alive [NBA] and stillborn [SB] piglets, caliper [CAL], average piglet birth weight [APBW], weaned piglets [WEAN], number of tail-enders [TEND].

The distributions of traits are shown in Figure 1, Figure 2 and Figure 3. Visually, the distributions for the mean (Figure 1) and slope traits (Figure 3) were similar. All standard deviation traits were right-tail skewed, reflecting some relatively high values (Figure 2). Most of the slope traits had a normal distribution (Figure 3). The left-tail skewness shows that the most common TEND count was small, while right-tail skewness in CAL shows some sows were more likely to have high than low body condition.

**Figure 4:**
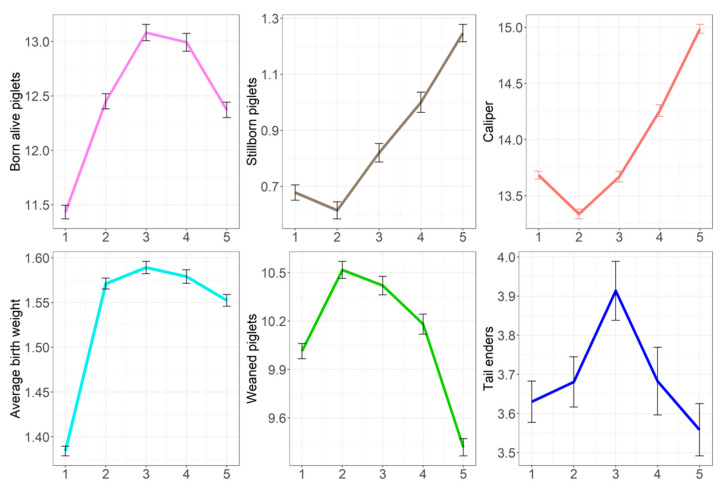
Least square means with SE for the number of born-alive and stillborn piglets, caliper, average piglet birth weight, weaned piglets, and number of tail-enders adjusted for contemporary group by parity group on X—axis (parity 1 = group 1, parity 2 = 2, parity 3 = 3, parity 4 = 4, > parity 4 = group 5).

Least square means by parity group for sow (NBA, SB, CAL) and maternal traits (APBW, WEAN, TEND) presented in Figure 4 demonstrate that both sow traits and maternal effects on progeny were not the same across parities. Changes with parity were non-linear. Sows in parity 3 had the highest number of both born-alive piglets and tail-enders. The level of NBA remained high in parity 4, while there was a reduction in the number of tail-enders. The lowest number of stillborn piglets, the highest average piglet birth weight, and the highest number of weaned piglets were in parity 2. However, pre-mating sow body condition was the lowest in parity 2, but not recorded pre-farrowing.

### 3.2. Variance Components

Heritability estimates for mean and slope traits were similar, except for tail-enders (Table 2). In contrast, heritability estimates for traits reflecting variability across parities (standard deviation traits) had negligible or very low estimates. The exceptions were sdTEND (h^2^: 0.06 ± 0.04) and sdCAL (h^2^: 0.05 ± 0.03), which did not significantly differ from zero. Overall, the highest heritability was estimated for piglet birth weight (mAPBW 0.35 ± 0.04 and APBWsl 0.38 ± 0.05) and caliper (mCAL 0.26 ± 0.04 and CALsl 0.27 ± 0.04). The number of tail-enders had similar heritability for the SD and mean trait definitions, whereas the slope was different (h^2^: 0.14 ± 0.04).

Mean and slope traits were genetically similar for tail-enders (r_A_: 0.64 ± 0.15), whereas variability (sdTEND) between parities was not (Table 3). Results generally indicated that the mean and slope traits were genetically the same, but were not reported here for all traits. The number of tail-enders was highly correlated with born alive piglets (r_A_: 0.71 ± 0.15). Variability in the number of tail-enders over parities (sdTEND) was also positively correlated with litter size, suggesting that progeny quality for sows with high litter sizes becomes more variable over time. Conversely, sows producing heavy piglets at birth (adjusted for total litter size) were less likely to have high number of tail-enders (r_A_: −0.83 ± 0.15). Slope traits showed the importance of preventing decline in birth weight with increased parity. A higher number of stillborn piglets was also genetically correlated with the higher mean number of tail-enders (r_A_: 0.43 ± 0.20). Further, sows with a low caliper score at mating had a higher number of tail-enders (r_A_: −0.57 ± 0.16, −0.52 ± 0.17) and negative correlations with litter size (r_A_: −0.34 ± −0.12, not shown).

## 4. Discussion

Selection for increased litter size is the most common strategy to increase the number of slaughter pigs produced per litter per sow. In addition to litter size, genetic improvement in piglet mortality or survival across different stages of lactation and growing periods can also occur, as demonstrated by other studies [22,23]. No study could be located that investigated the genetic potential of a sow to produce pigs with satisfactory performance to meet target weights at the required age (i.e., growth). Although target weights vary across countries [24], the common goal is a focus on growth and high outputs, e.g., kg per sow per year, which reflects growth, number of finishers per sow, and the required slaughter weight. This goal is addressed generally in breeding programs by having complex breeding objectives with selection criteria that reflect each sow’s reproductive traits, progeny survival, and progeny growth characteristics. The present study suggests that selection for increased litter size generally results in more pigs of lower quality at slaughter (i.e., poorer weight for age, increased losses) when assessed over multiple parities. This outcome is modified if piglet birth weights can be sustained, but, despite including progeny growth in breeding objectives, the high number of tail-enders is still evident.

The trait ‘tail-enders’ could encompass any or all of: piglets euthanized as runts at birth, piglets delayed in growth relative to their contemporaries, pigs sent to the slaughterhouse with light weights or at a later age than the targeted age at slaughter, or those accidentally missing. The number of pigs that never reach the targeted end weight (or were not selected as future parents) incur more costs as pigs are held on a farm longer and penalties in the slaughterhouse could potentially be larger. Consequently, the tail-ender trait represents a complex and an economically important production issue, yet there is a lack of knowledge about the genetic background. Maternal breeding objective traits, with emphasis on direct and maternal effects on growth and progeny performance, indirectly addressed the tail-enders trait. The high number of tail-enders suggests that this was potentially not entirely effective. The sire contribution to piglet growth is also important, but each sire was mated at random to many dams. Many sires were represented by progeny that were tail-enders, as expected. In this population, TEND was partially influenced by sub-optimal health status. In other populations, TEND could vary due to different lines, farms, seasons, health status, or recording errors, which needs to be further investigated. Recording tail-enders could, therefore, be beneficial in identifying why reduced performance was present in particular litters. There are, however, some deficiencies in trait definition by inference. The animals recorded as dead were not a part of this trait in order to avoid double counting (e.g., if a breeding program contained survival traits already). A proportion of animals were also not recorded by accident. Some examples of non-recording by accident include tag or identity loss, record entry failure, and failure to record all mortalities accurately. These factors all increase the inferred number of tail-enders, but such errors should not be systematic with respect to litters. Therefore, this trait definition is proposed in the first instance, while recognizing potential deficiencies of this approach.

Mean TEND, treated as a trait of the sow, was more variable but less heritable than mNBA, supporting a combination of maternal and additional external influences in outcomes for progeny to slaughter age. A high heritability for the slope indicated that there is a potential to alter this trait genetically. The moderate-to-high genetic correlations between the number of born-alive piglets and the number of tail-enders suggest that not adequately accounting for this trait in the breeding program could result in an undesirable increase in the number of slow-growing pigs, runts, pigs with health problems, and non-selected pigs. Given that as the litter size increases, the birth weight decreases [25], this potentially resulted in more tail-enders, which is a welfare concern. However, selection for increased birth weight was already a selection criterion in this population. The positive correlation for the slope in the number of stillborn piglets and the mean number of tail-enders, along with the strong negative correlation with birth weight, demonstrated the importance of including these traits in breeding programs.

The importance of sow body condition on performance, welfare, and the farm productivity is well known. Maintaining optimal sow body condition starts with gilts, as the optimal body condition is closely related to the lifetime performance [26,27]. This practice continues throughout all stages during the sow’s productive lifetime. Sub-optimal body condition requires adjustments of the feed allocation, typically performed during the gestation period. Other studies have shown that unfulfilled sow nutrient demands lead to poor performance, such as an elevated number of stillborn piglets, low number of weaned piglets, or early culling [28,29]. These have negative consequences on piglet survival and growth, leading to a higher number of days from birth to market. Based on results observed for TEND following low CAL and high NBA (Figure 4), our study suggests that sow nutrient demands may be unfulfilled and this also leads to more tail-enders. However, the impact of a sow’s variability in body condition throughout the lifetime on the overall performance, the mortality of piglets, and the number of pigs that are culled earlier due to the poor performance (i.e., growth) is unknown. Sows with a lower mean or slope for caliper pre-mating were genetically more likely to have a high number of tail-enders, suggesting maintaining appropriate (higher) sow condition is important to reduce the occurrence of poor quality slaughter progeny. Low body condition in sows is frequently associated with poor intake [30] and higher litter size.

For individual sows, maternal performance alters with age. In this study, sow performance over parities was represented as a mean, standard deviation, or slope for each trait, which characterized variability and trends in maternal performance over parities. Sows with a higher mean had better performance over their lifetime (for traits such as born alive or weaned piglets, or average piglet birth weight), whereas the higher standard deviation reflected increased variation across parities. The information provided by mean was very similar to that provided by slope (except for tail-enders). The trajectory over the sow’s lifetime was represented by slope. Estimates of both mean and slope were affected by the pattern of performance change over parities, reflecting both increases and decreases with parity.

Genetic correlations indicated that mean and slope definitions for traits were essentially supplying the same information. The higher mean in traits increased the performance over the lifetime, whereas slope traits identified whether a sow had increasing or declining performance over time. This potentially could not be seen if a sow had, for example, a very high number of born alive piglets in first parity followed by low-to-moderate numbers of piglets in subsequent parities. Further, low-to-moderate correlations for litter size with the number of tail-enders (mean, slope, standard deviation) indicated that this trait was genetically different to litter size traits observed over parities.

Variability in performance across parities (SD traits) had a large coefficient of variation, demonstrating that the within-sow variability and the variability amongst sows were very high when traits representing performance across parities were assessed. Variance components for the range in total born piglets across parities [31] or born-alive piglets and average piglet birth weight [32] were similar to the present study, demonstrating the usefulness in the trait definitions across different production systems and genetic lines. Very low heritability estimates for SD traits demonstrated that variation in performance across parities generally occurred at random and reducing this form of variation with selection would be challenging.

## 5. Conclusions

Accounting for the sow’s influence on the number of tail-enders in breeding programs could assist with producing more high-quality slaughter pigs. Genetically and phenotypically, this number correlated favorably (e.g., CAL, ABWT) and unfavorably (NBA) with other reproductive and maternal performance traits. The number of poorer quality piglets that remained in the pig production systems to slaughter age was both variable and heritable. We suggest that maternal line breeding goals and management may not adequately address this issue. The trait definition used in this study where the number of absent pigs was treated as a tail-ender value potentially has deficiencies. Further investigation into alternative trait definitions in other lines and populations should also be investigated.

Each sow or maternal trait in this study was expressed as mean, slope, and standard deviation, representing the variability in change over parities. Traits expressed as mean and slope were heritable and genetically similar. Variation across parities occurred at random for several traits (i.e., SD traits were generally not heritable), whereas slope and mean traits had the potential to be altered with selection.

## Figures and Tables

**Table 1:** Descriptive statistics of maternal performance traits by parity. Characteristics of the sow: number born alive (NBA), stillborn piglets (SB), caliper increments (CAL). Characteristics with maternal effect on performance: average piglet birth weight (APBW), number of weaned piglets (WEAN), number of tail-enders (TEND). Measurements across parity: mean (m*), standard deviation (sd*), and slope (*sl).

Trait ^1^	N Records	Mean (SD) ^2^	Min–Max	IQR 0.25–0.75 ^3^	CV ^4^
Traits by parity					
NBA	13,976	12.2 (3.00)	0–23	10–14	24.6
SB	13,976	0.89 (1.31)	0–18	0–1	1.48
CAL	9033	14.1 (2.13)	6–22	13–16	15.1
APBW	8495	1.52 (0.24)	0.69–2.48	1.36–1.65	15.6
WEAN	13,209	10.2 (2.21)	0–26	9–12	21.7
TEND	6462	3.89 (2.56)	0–18	2–5	65.9
Across parity traits					
mNBA	3592	12.0 (2.00)	1–18.3	11–13.3	16.6
mSB	3592	0.84 (0.82)	0–7	0.25–1.14	98.3
mCAL	3338	13.9 (1.50)	7–20	13–15	10.7
mAPBW	3532	1.51 (0.20)	0.74–2.48	1.38–1.62	13.1
mWEAN	3538	10.2 (1.71)	0–20	9.5 – 11.2	16.7
mTEND	3299	3.82 (2.10)	0–18	2.25 – 5.00	54.9
sdNBA	2922	2.47 (1.29)	0–10.6	1.53–3.20	52.3
sdSB	2922	0.94 (0.77)	0–8.96	0.52–1.26	81.9
sdCAL	2468	1.51 (0.86)	0–5.66	0.89–2.06	56.8
sdAPBW	2291	0.18 (0.11)	0–0.86	0.096–0.227	63.2
sdWEAN	2854	1.76 (1.17)	0–9.90	1.00–2.12	66.7
sdTEND	1733	2.06 (1.38)	0–9.90	1.00–2.83	67.1
NBAsl	2922	0.006 (0.157)	−0.686–0.663	−0.083–0.095	NA
SBsl	2922	0.016 (0.098)	−0.145–0.751	−0.044–0.052	NA
CALsl	2466	0.053 (0.210)	−0.634–0.779	−0.076–0.187	NA
APBWsl	2291	0.003 (0.016)	−0.057–0.062	−0.006–0.012	NA
WEANsl	2854	−0.011 (0.075)	−0.484–0.313	−0.042–0.033	NA
TENDsl	1733	−0.003 (0.033)	−0.173–0.163	−0.020–0.015	NA

^1^ Units: NBA (piglets/litter), SB (piglets/litter), CAL (increments), APBW (average kg per litter), WEAN (piglets/litter), TEND (pigs/litter); ^2^ standard deviation (SD); ^3^ interquartile range (IQR); ^4^ coefficient of variation (CV, %); NA (not applicable).

**Table 2:** Estimates of variance components and heritability with standard errors in brackets (SE) for maternal performance traits: mean, standard deviation, and slope phenotypes obtained per sow from univariate models.

Trait ^1^	Additive Genetic Variance	Residual Variance	Heritability
mNBA	0.44 (0.11)	3.38 (0.11)	0.12 (0.03)
mSB	0.07 (0.01)	0.61 (0.02)	0.10 (0.03)
mCAL	0.53 (0.09)	1.53 (0.07)	0.26 (0.04)
mAPBW	0.01 (0.002)	0.02 (0.001)	0.35 (0.04)
mWEAN	0.23 (0.07)	2.58 (0.07)	0.08 (0.02)
mTEND	0.25 (0.10)	3.75 (0.12)	0.06 (0.02)
sdNBA	3.9 × 10^−8^ (0.00)	1.63 (0.04)	0.00 (0.00)
sdSB	5.3 × 10^−5^ (0.0053)	0.56 (0.02)	0.0001 (0.01)
sdCAL	0.04 (0.02)	0.68 (0.03)	0.05 (0.03)
sdAPBW	2.8 × 10^−4^ (2.8 × 10^−4^)	0.01 (4.2 × 10^−4^)	0.03 (0.02)
sdWEAN	1.4 × 10^−6^ (0.00)	1.36 (0.04)	0.00 (0.00)
sdTEND	0.11 (0.06)	1.67 (0.08)	0.06 (0.04)
NBAsl	3.1 × 10^−3^ (7.7 × 10^−4^)	2.2 × 10^−2^ (8.2 × 10^−4^)	0.13 (0.03)
SBsl	1.3 × 10^−3^ (3.0 × 10^−4^)	8.0 × 10^−3^ (3.1 × 10^−4^)	0.14 (0.03)
CALsl	1.1 × 10^−2^ (1.9 × 10^−2^)	2.8 × 10^−2^ (1.5 × 10^−3^)	0.27 (0.04)
APBWsl	9.1 × 10^−5^ (1.3 × 10^−5^)	1.5 × 10^−4^ (9.4 × 10^−6^)	0.38 (0.05)
WEANsl	5.4 × 10^−4^ (1.5 × 10^−4^)	4.8 × 10^−3^ (1.8 × 10^−4^)	0.10 (0.03)
TENDsl	1.6 × 10^−4^ (4.7 × 10^−5^)	9.4 × 10^−4^ (4.9 × 10^−5^)	0.14 (0.04)

^1^ Trait abbreviations: number born alive (NBA), stillborn piglets (SB), caliper increments (CAL), average piglet birth weight (APBW), number of weaned piglets (WEAN), number of tail-enders (TEND). Across-parity measurements: mean (m*), standard deviation (sd*) and slope (*sl).

**Table 3:** Genetic (r_A_) and phenotypic (r_P_) correlations with standard errors in brackets (SE) between tail-enders (TEND) and the maternal traits.

Traits ^1^	Correlation	mTEND	sdTEND	TENDsl
mNBA	r_A_	0.71 (0.15)	0.46 (0.26)	0.37 (0.18)
	r_P_	0.34 (0.02)	0.13 (0.03)	0.07 (0.03)
mSB	r_A_	NC	0.45 (0.29)	−0.30 (0.19)
	r_P_		0.04 (0.03)	−0.17 (0.03)
mAPBW	r_A_	NC	−0.10 (0.21)	NC
	r_P_		−0.04 (0.03)	
mWEAN	r_A_	0.20 (0.24)	−0.36 (0.31)	0.03 (0.21)
	r_P_	0.09 (0.02)	−0.02 (0.03)	0.06 (0.03)
mTEND	r_A_	NA	0.17 (0.37)	0.64 (0.15)
	r_P_		0.49 (0.02)	0.76 (0.01)
mCAL	r_A_	−0.57 (0.16)	−0.13 (0.24)	−0.29 (0.16)
	r_P_	−0.03 (0.02)	0.04 (0.03)	−0.03 (0.03)
sdTEND	r_A_	0.17 (0.37)	NA	0.25 (0.28)
	r_P_	0.49 (0.02)	NA	0.27 (0.03)
sdCAL	r_A_	−0.38 (0.30)	−0.42 (0.41)	0.12 (0.28)
	r_P_	−0.04 (0.02)	−0.01 (0.03)	0.009 (0.03)
NBAsl	r_A_	NC	0.31 (0.26)	0.31 (0.18)
	r_P_		0.08 (0.02)	0.08 (0.03)
SBsl	r_A_	0.43 (0.20)	0.47 (0.26)	−0.26 (0.18)
	r_P_	−0.0008 (0.02)	0.03 (0.02)	−0.14 (0.02)
APBWsl	r_A_	−0.83 (0.15)	−0.10 (0.21)	NC
	r_P_	−0.09 (0.02)	−0.008 (0.03)	
WEANsl	r_A_	0.32 (0.23)	−0.10 (0.28)	0.28 (0.21)
	r_P_	0.04 (0.03)	−0.03 (0.02)	0.05 (0.02)
CALsl	r_A_	−0.52 (0.17)	−0.28 (0.26)	−0.29 (0.17)
	r_P_	−0.03 (0.02)	0.03 (0.03)	−0.02 (0.03)

^1^ Trait abbreviations: number born alive (NBA), stillborn piglets (SB), caliper increments (CAL), average piglet birth weight (APBW), number of weaned piglets (WEAN), number of tail-enders (TEND). Across-parity measurements: mean (m*), standard deviation (sd*), and slope (*sl); NA = not applicable; NC = not converged.

## Data Availability

The data supporting reported results may be available upon request from the corresponding author.
